# Omega Rhodopsins: A Versatile Class of Microbial Rhodopsins

**DOI:** 10.4014/jmb.1912.12010

**Published:** 2020-04-02

**Authors:** Soon-Kyeong Kwon, Sung-Hoon Jun, Jihyun F. Kim

**Affiliations:** 1 Division of Life Science, Gyeongsang National University, Jinju 52828, Republic of Korea; 2 Electron Microscopy Research Center, Korea Basic Science Institute, Cheongju 8119, Republic of Korea; 3 Department of Systems Biology, Division of Life Sciences, and Institute for Life Science and Biotechnology, Yonsei University, Seoul 0722, Republic of Korea

**Keywords:** 3 omega motif, actinorhodopsin (ActR), chloride pump rhodopsin (ClR), sodium pump rhodopsin (NaR), xanthorhodopsin (XR)

## Abstract

Microbial rhodopsins are a superfamily of photoactive membrane proteins with the covalently bound retinal cofactor. Isomerization of the retinal chromophore upon absorption of a photon triggers conformational changes of the protein to function as ion pumps or sensors. After the discovery of proteorhodopsin in an uncultivated γ-proteobacterium, light-activated proton pumps have been widely detected among marine bacteria and, together with chlorophyll-based photosynthesis, are considered as an important axis responsible for primary production in the biosphere. Rhodopsins and related proteins show a high level of phylogenetic diversity; we focus on a specific class of bacterial rhodopsins containing the ‘3 omega motif.’ This motif forms a stack of three non-consecutive aromatic amino acids that correlates with the B–C loop orientation and is shared among the phylogenetically close ion pumps such as the NDQ motif-containing sodiumpumping rhodopsin, the NTQ motif-containing chloride-pumping rhodopsin, and some protonpumping rhodopsins including xanthorhodopsin. Here, we reviewed the recent research progress on these ‘omega rhodopsins,’ and speculated on their evolutionary origin of functional diversity.

## Introduction

**Fig. 1:**
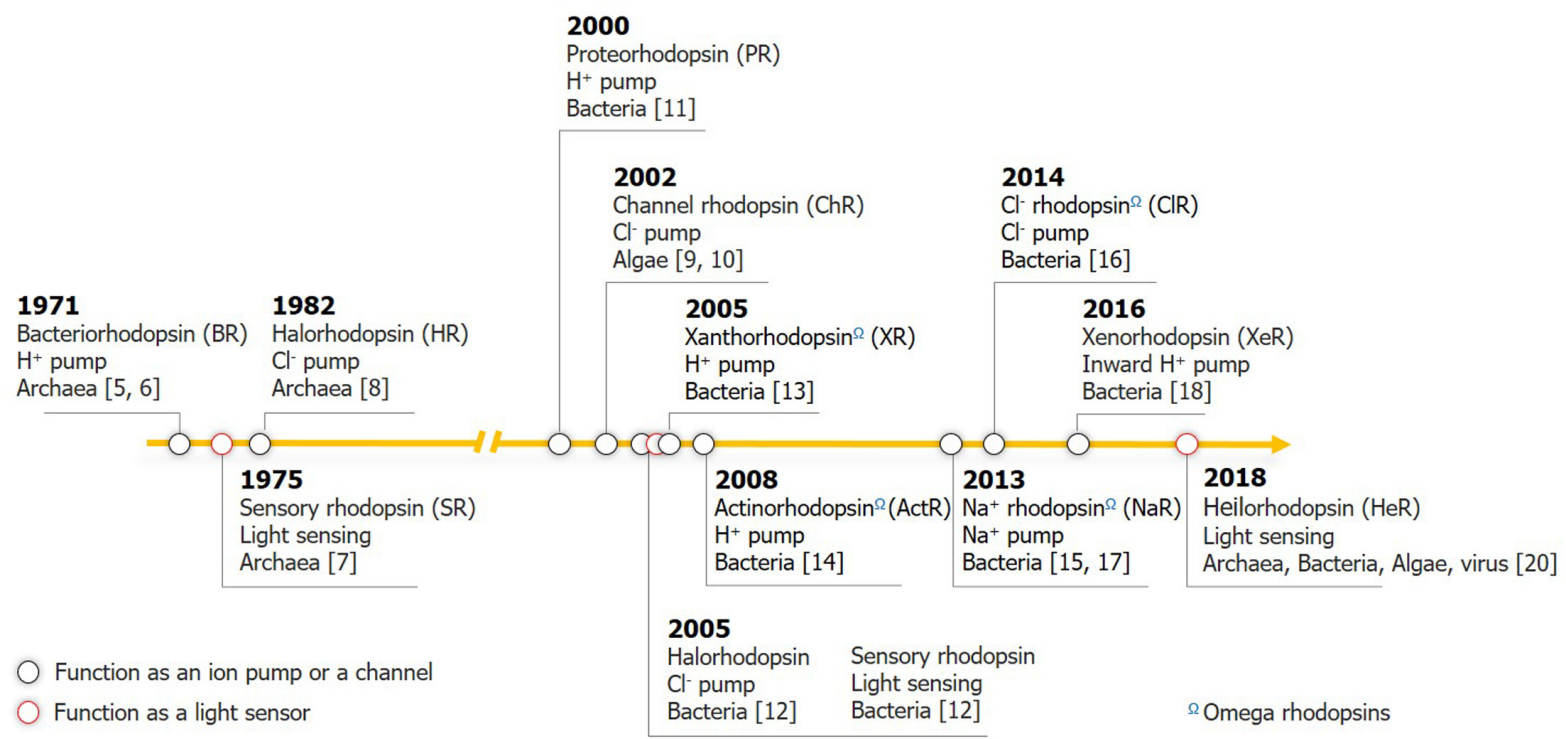
Key milestones in microbial rhodopsin research. Rhodopsins that function as ion pumps or channels are shown in black circles; sensory rhodopsins are in red. Omega rhodopsins are indicated Ω.

Microbial rhodopsins, termed as ‘type I rhodopsins’ to distinguish them from the G-protein coupled receptor superfamily (type II rhodopsins) that comprises several opsin families, are photoactive proteins consisting of seven transmembrane (TM) domains. These proteins mainly act as light-driven ion pumps or sensors with the configurational change in incorporated all-*trans* retinal [1-3]. This simple light-harvesting system seems prehistoric to the chlorophyll-based photosynthetic machinery, both of which are responsible for primary production in the photic zone and are efficient facilitators of solar energy influx into the biosphere and biogeochemical cycle [4]. A brief history of microbial rhodopsin research is presented in Fig. 1.

The first discovered and most widely understood rhodopsin is bacteriorhodopsin (BR), which was found from Archaea in the 1970s [5, 6], and its ability to translocate the proton ion that results from ATP biosynthesis was further elucidated [5, 6]. The subsequent discovery of an inward chloride ion pump, halorhodopsin (HR), and sensory rhodopsin (SR), which is responsible for phototactic responses, have broadened the spectra of haloarchaeal rhodopsins [7, 8]. Additionally, channel rhodopsin (ChR), a light-gated cation channel, was discovered in eukaryotic green algae [9, 10]. Members of ChR are adopted as successful tools for optogenetics, wherein they react to light and allow the influx of positive ions to act as an ‘on’ switch. In contrast, HR, which translocates negatively charged chloride ion, is widely used for inactivation in neuroscience.

Research on microbial rhodopsins has markedly expanded along with the discovery of proteorhodopsin (PR) from Proteobacteria through a marine metagenomic analysis [11]. PR shares structural and functional similarities with the archaeal BR; however, its presence in Bacteria led to the expectation that rhodopsin may have a greater impact on the marine ecosystem. Subsequently, three haloarchaeal-type rhodopsin genes including those of an HR and two SRs were detected in the genome of *Salinibacter ruber*, which was isolated from a salt-crystallizer pond [12]. From this isolate, another interesting proton-pumping rhodopsin, xanthorhodopsin (XR), with two chromophores was identified, which interacts with the retinal and carotenoid salinixanthin [13]. Moreover, sequences of a novel group of rhodopsins were discovered via metagenomic mining of several non-marine aquatic environments; these novel clades of green light-dependent proton-pumping rhodopsins are collectively called ‘actinorhodopsins’ (ActRs) [14].

In the 2010s, wing to mind-boggling developments in genomics, new clades of bacterial rhodopsins had been continuously discovered. A sodium-pumping rhodopsin (NaR) with the NDQ motif in its active site and a chloride-pumping rhodopsin (ClR) with the NTQ motif were reported in turn from marine flavobacteria [15-17]. Xenorhodopsin, a natural inward proton pump, was also characterized, which is unique since other previously reported unidirectional cation pumps are all outward [18]. As the newest members of the bandwagon, viral-type proton-pumping rhodopsins were retrieved from giant viruses of eukaryotic algae through single-cell metagenomics [19]. More recently, heliorhodopsin with its speculated function of light-sensing activity was found to be widespread globally across the three domains of life and algal viruses [20]. All of these efforts have profoundly expanded the spectrum of type I microbial rhodopsins and phylogenetically related rhodopsins.

Among the microbial rhodopsins, this review focuses on the ion-translocating rhodopsins that comprise the ‘3 omega motif ’ that forms a stack of three non-consecutive aromatic amino acids arising from the TM helices A and B, and the B–C loop. We refer to the class of microbial rhodopsins containing this structural motif as ‘omega rhodopsins.’ We review recent developments in elucidating the functions and characteristics of this rhodopsin group. A thorough understanding of this rhodopsin class will provide insights into the evolution of 7-TM proteins with consideration to their phylogenetic distribution and functional or ecological diversification.

## Recognition of the ‘3 Omega Motif’

**Fig. 2:**
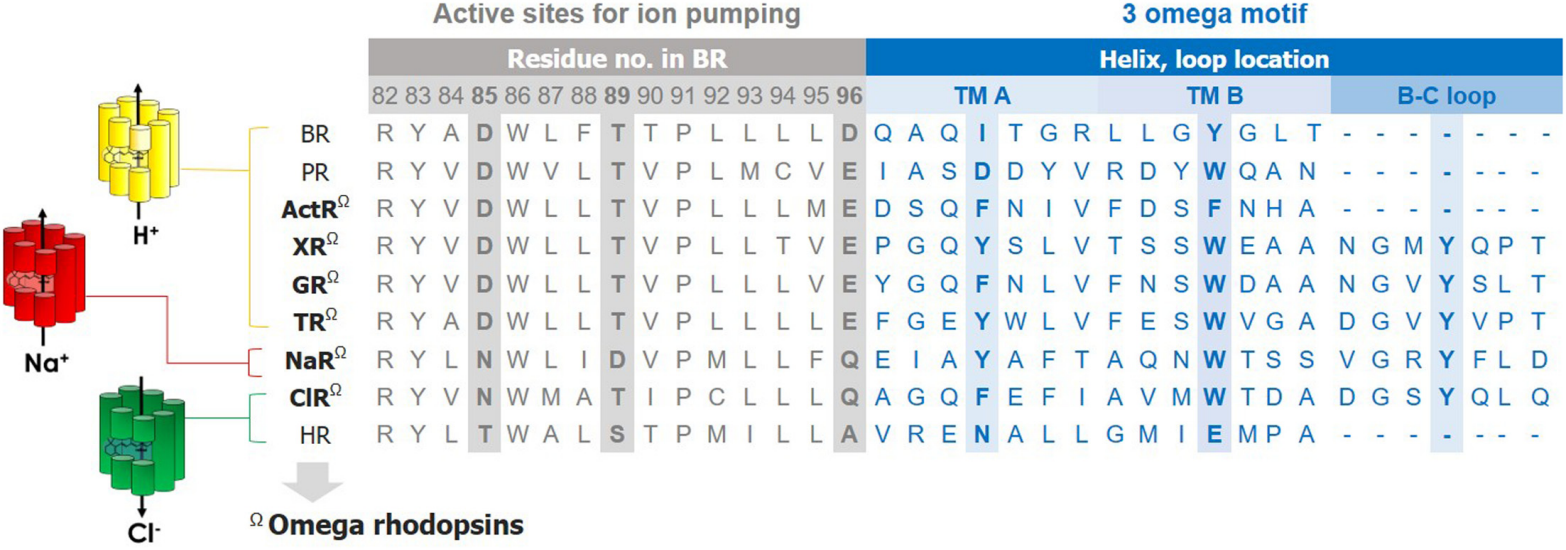
Characteristic features of type I microbial rhodopsins with the ‘3 omega motif.’ Sequence alignment in the active sites and 3 omega motif. The representative amino-acid sequences of each rhodopsin adopted from *Halobacterium salinarum* NRC-1 (BR), *Dokdonia* sp. MED134 (PR), *Actinobacterium*_MWH_Uga1 (ActR), *Salinibacter ruber* DSM 13855 (XR), *Gleobacter violaceus* PCC7421 (GR), *Thermus thermophilus* JL-18 (TR), *Dokdonia erikasta* NBTC 100814 (NaR), *Nonlabens marinus* S1-08 (ClR), *Halobacterium salinarum* NRC-1 (HR).

**Fig. 3:**
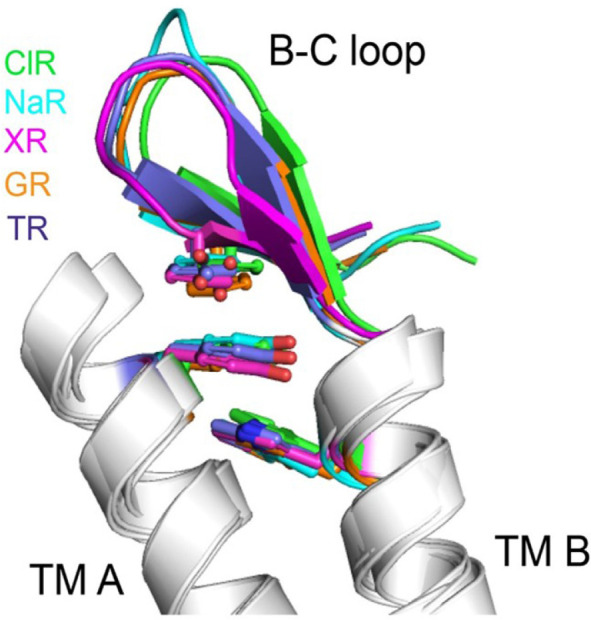
Three-dimensional structures of the 3 omega motifs. NDQ motif-containing sodium-pump rhodopsin, NaR (cyan, PDB ID: 3X3B); NTQ motif-containing chloride-pump rhodopsin, ClR (green, PDB ID: 5G28); xanthorhodopsin, XR (magenta, PDB ID: 3DDL); *Gleobacter* rhodopsin, GR (orange, PDB ID: 6NWD); thermophilic rhodopsin, TR (blue, PDB ID: 5AZD).

We noticed the presence of the 3 omega motif during the structural analysis of a ClR from *Nonlabens marinus* S1-08. This motif comprises three nonconsecutive aromatic amino acids located at Phe15 in TM A, Trp72 in TM B, and Tyr83 in the B-C loop. The side chains of these aromatic amino acids are stacked upon one another, tethering the B–C loop in the direction of TMs A and B (Figs. 2 and 3). The presence of these aromatic amino-acid residues correlates with the orientation of the B–C loop. This distinctive feature is conserved among ClRs, NaRs, and XRs, which are evolutionarily closer to one another than to the other microbial rhodopsins [21].

**Fig. 4:**
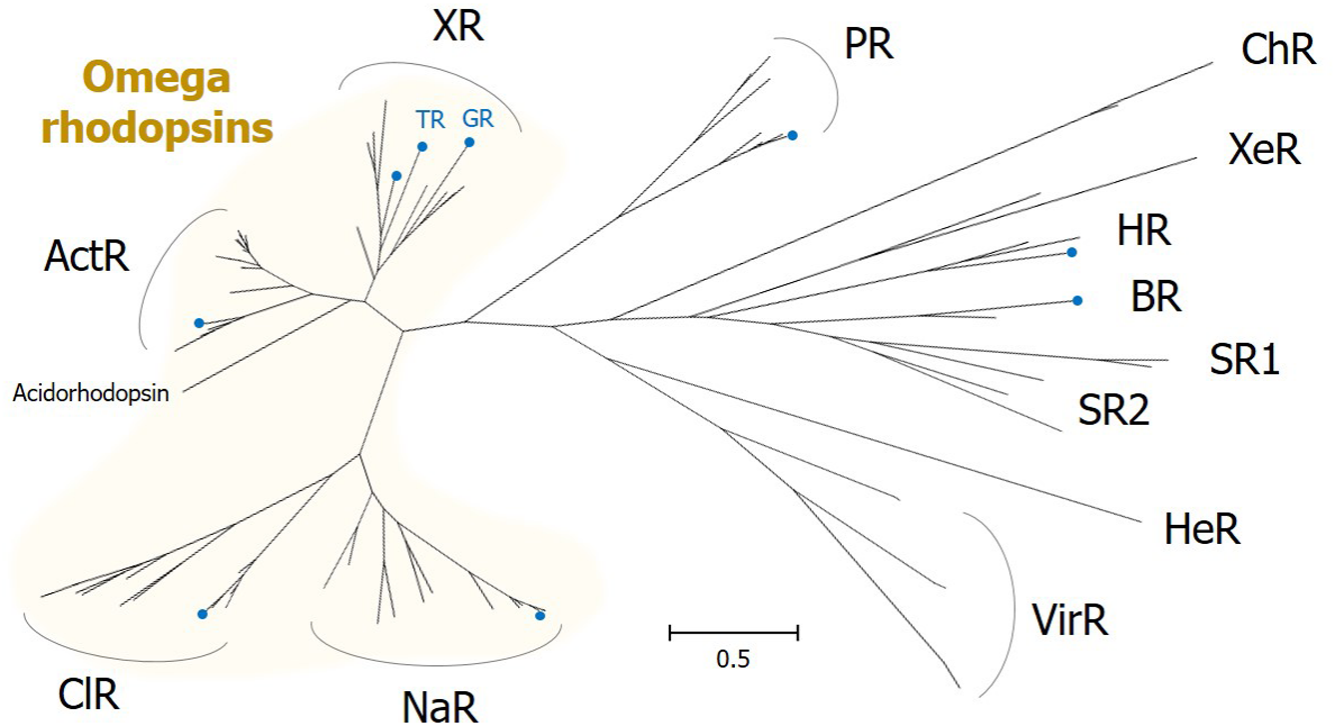
Phylogenetic relationships between omega rhodopsins and other microbial rhodopsin families. The maximum-likelihood method using the Jones-Taylor-Thornton matrix-based model of amino acid substitution rates with empirical amino acid frequencies and the gamma model was used. Representative microbial rhodopsins were aligned by MUSCLE. Evolutionary analyses were conducted in MEGA X. Blue-colored circles on the tip of some branches indicate the rhodopsins listed in Fig. 2. NDQ motif-containing sodium-pump rhodopsin, NaR; NTQ motif-containing chloride-pump rhodopsin, ClR; xanthorhodopsin, XR; *Gleobacter* rhodopsin, GR; thermophilic rhodopsin, TR; actinorhodopsin, ActR; proteorhodopsin, PR; bacteriorhodopsin, BR; halorhodopsin, HR; channel rhodopsin, ChR; sensory rhodopsin, SR; xenorhodopsin, XeR; heliorhodopsin, HeR; viral rhodopsin, ViR.

ActR is a group of green light-dependent, outward proton-pumping rhodopsins and is phylogenetically close to XR (Fig. 4). Although the protein structure has not yet been elucidated for any of the members, alignment of protein sequences revealed that the amino acids with aromatic rings are conserved in TM A and TM B, but not in the B-C loop (Fig. 2). Even without the presence of the third aromatic functional group, the stacking of two aromatic rings in TMs A and B could be sufficient to contribute to the stability of the two helices and the whole protein. Thus, we classify ActR as an omega rhodopsin in this review.

The function of the 3 omega motif is yet to be clearly elucidated. The ﬂuorescent thermal stability assay of mutants, wherein the aromatic amino acids were substituted with alanine, revealed that mutants were slightly unstable compared with the wild-type ClR [21]. Moreover, Morizumi *et al*. elucidated an XR-family rhodopsin from the cyanobacterium *Gleobacter violaceus*, and they claimed that the ‘3 omega motif ’ affects oligomerization rather than proton pumping [22]. As Shibata *et al*. had determined, NaRs and the XR family of proton pumps including *Gleobacter* rhodopsin (GR) form only pentameric oligomers [23], and these proton pumps were reclassified based on the oligomerization propensities and conserved structural features such as extended helices A and B, the 3 omega motif, and the flipped B-C loop [22].

We hypothesize that the structural rigidity and stability of TM helices A and B generated by stacked aromatic rings could support the diagonal flexibility and freedom of other transmembrane columns, resulting in functional innovation with respect to the extended ion specificity and absorption spectrum during the evolution of rhodopsins in this class.

## Functional Diversity of Omega Rhodopsins

Omega rhodopsins have a wide functional versatility in terms of ion specificity and absorption spectrum. Fig. 4 presents the phylogenetic relationships within or between omega rhodopsins and other type I rhodopsins. Phylogenetic analysis indicates that this class of microbial rhodopsins is evolutionally distinct from the others, thus constituting a unique phylogenetic clade. We will summarize the brief history and recent studies on omega rhodopsins according to their phylogenetic affiliation and translocated ions, namely H^+^, Na^+^, and Cl^-^ ions.

## The XR Family of Proton Pump Rhodopsins

The most representative family of proton pumps among the omega rhodopsins is the XRs. The photocycle and functional amino acids in the ion transfer pathway in XR remain similar to the other proton pumps, such as PR or BR, but their structure differs considerably. In addition to retinal, XR is associated with a particular carotenoid antenna molecule, salinixanthin, whose function is light-harvesting and acting as an energy provider for the acceptor, retinal [13]. The structure of the XR initially found in *S. ruber* M31 reveals 12 residues for carotenoid binding [24]. Seven out of 12 residues are conserved in GR, which is highly similar to XR and binds to echinenone, comprising a keto-ring similar to salinixanthin [25]. This carotenoid expands the range of wavelengths. The range of absorption extends from 560 nm with XR alone to 457, 486, and 560 nm by coupling to the carotenoid salinixanthin [13]. Marked differences between XR and other proton pumps were observed in the formations of the main chain. The length, tilt, and rotation of helices differ considerably, particularly that of helix A, and the unique form of B-C loop of XR results in a large cleft that extends far into the interior and brings functional residues, buried in other rhodopsins [24]. The retinal conformation around the protonated Schiff base is more similar to the other families of omega rhodopsins and ChRs rather than those of BRs or PRs [21].

A proton pump was found in the hyperthermophilic bacterium *Thermus thermophilus* JL-18, which inhabits hot springs at around 75°C, and was named ‘thermophilic rhodopsin’ (TR) [26]. TR exhibited a high similarity in the amino-acid sequence and structural features with XR. Critical residues for binding to the carotenoid antenna are conserved in XR [27], and *T. thermophilus* contains gene sets for thermozeaxanthin, which shares a higher similarity with salinixanthin [28]. Furthermore, the formation of a novel antenna complex between TR and the carotenoid salinixanthin was confirmed [29]. TR also comprises the 3 omega motif and the flipped B-C loop that are distinguishable from PRs or BRs [22, 27]. Notably, TR differs from XR in its extremely high thermal stability achieved by a smaller number of intramolecular hydrogen bonds, a larger number of hydrophobic residues, and the interactions of aromatic residues within the protein and with the membrane lipid molecules. Especially, the Leu211-Pro212-Gly213-Gly214 sequence in the F-G loop is presumed to contribute its thermal stability decisively [27].

GR is a proton pump found in *G. violaceus* PCC 7421, which was isolated from a calcareous rock [30]. This cyanobacterium uniquely lacks thylakoid membranes, and its photosynthetic machinery exists in the cytoplasmic membrane with GR [30, 31]. Its efficient proton pumping and rapid photocycle were experimentally confirmed, suggesting that it could compensate for the low energy production due to the lack of chlorophyll-dependent photosynthesis [31]. GR has the ability to bind light-harvesting antenna comprising 4-keto headgroup, carotenoid salinixanthin from *S. ruber* or echinenone from *G. violaceus* [25], thus leading to the efficient energy transfer from carotenoid to the retinal [32, 33]. Also, it is reported that the presence of salinixanthin accelerates the retinal–protein covalent bond formation [34]. Determination of the structure of GR revealed that its overall structure is homologous to XR. It possesses the 3 omega motif and a proton transfer pathway similar to that of XR, but tighter packing of transmembrane helices at the extracellular side is a distinguishing feature of GR [22].

## Actinorhodopsin Proton Pumps

ActRs are most closely related to XR in the phylogenetic tree (Fig. 4). This group of rhodopsins was initially found by mining the metagenomic data of non-marine samples [14, 35]. Genes that are present in the metagenomic assemblies in this rhodopsin clade are linked to the sequenced actinobacterial genomes, and therefore it is termed ‘ActR’ [14]. ActRs have been excavated from numerous mixed cultures containing Actinobacteria and environmental DNA samples from various freshwater environments, thus revealing that these genes are dispersed globally.

ActRs in freshwater comprise the three phylogenetic groups LG1, LG2, and PCL1, and these sequences were also reported from freshwater lakes, estuaries, and hypersaline lagoon ecosystems [14]. LG1 group is split into subgroups, and LG1-A groups are encoded by acI, which is the most abundant actinobacterial lineage among the freshwater inhabitants, whereas genes of the LG1-B group are carried by the Luna lineage [36, 37]. The ability of ActR to pump proton in the native cells was confirmed by supplementing with exogenous retinal [38] or innate retinal [39]. ActR in *Rhodoluna lacicola* belonging to the acI lineage was active only if the exogenous retinal was supplied, suggesting that the cells lacked the retinal synthesis machinery [38]; in contrast, the Luna bacterium *Candidatus* Rhodoluna planktonica encodes a putative retinal-synthesizing system [39]. The biosynthetic route for retinal and carotenoids, which are necessary for the ActR function is elucidated in the acI lineage [40]. ActRs also contain the glycine residue for binding ketolated antenna carotenoids. Although ActRs are known to be widespread in freshwater Actinobacteria, they are almost completely absent from the marine environment. Nevertheless, a rhodopsin clade that is highly related to freshwater ActRs was discovered from a marine actinobacterial genome obtained by marine metagenome data assembly [41]. Subsequent searches against the marine metagenome datasets yielded more similar sequences, and this cluster was designated as ‘acidorhodopsin’ after the order *Acidimicrobiales*. More marine ActR sequences were obtained from a metagenomic fosmid library from the Red Sea by using a functional rhodopsin screening system that enables the measurement of proton-pumping activity [42].

## NDQ Motif-Containing Sodium Pump Rhodopsins

In 2013, a novel class of microbial rhodopsins possessing Asn and Gln at the positions of the proton acceptor and donor residues of BR was found in *Nonlabens (Donghaeana) dokdonensis* DSW-6 and other bacterial genomes [15]. In *N. dokdonensis*, the gene expression of NQ motif-containing rhodopsin was induced by increasing the concentration of NaCl in the culture media, and rhodopsins with the NQ motif are frequently found in hypersaline environmental metagenomic datasets. The rhodopsin KR2, the second rhodopsin of *Dokdonia eikasta* (synonym *Krokinobacter eikastus*), which forms the same phylogenetic clade with *N. dokdonensis* rhodopsin with the NQ motif, was proved to be an outward sodium pump (thus called ‘NaR’) [17]. Their functional group, NQ, was redefined as the NDQ motif after the discovery of a chloride pump with the NTQ motif [16].

Although NaR is optimized for sodium ion pumping, it can also pump lithium and even convert to a proton pump in the absence of sodium or lithium ions. However, its proton-pumping photocycle is slower than that during sodium pumping, and its efficiency is considerably lower than that of the typical proton pumps [17]. Extensive studies on ion selectivity using KR2 revealed that the rate constant of ion uptake is dependent on ion concentration, and NaR pumps sodium under physiological conditions in which the sodium concentration is significantly greater than that of the proton [43-45]. The cation tuning exhibited by NaR is unique among microbial rhodopsins. In the KCl solution, NaR acts as a proton pump, whereas it functions as a sodium pump in the NaCl solution. Its cation selectivity is based on the different helical movements in the presence of K^+^ or Na^+^ [46]. Switching mechanism from sodium pumping to proton pumping at acidic pH was proposed by Kovalev *et al*.[47]. The decreased pH leads to the protonation of Asp 116 and triggers the rearrangement for reducing the Schiff base cavity in compact structures, thereby allowing the passage of smaller proton ions [47]. Moreover, the biophysical properties including ion selectivity of various NaRs were measured, and differences were observed in the sodium selectivity among NaRs [48].

The crystal structures of NaR under neutral and acidic conditions, and three different acid pHs in the monomeric state or pentameric states, were reported [49]. Along with detailed biochemical characterization and mutagenesis, both studies successfully figured out the sodium-translocation mechanism. In particular, the NaR structure provides the first clue how positively charged sodium ion, which is unable to bind covalently to the Schiff base, achieves transport across the conduit. Unlike proton pumps, NaR contains distinguishable structures including N-terminal helix capping the ion-release cavity and loop B-C over a hydrophilic cavity to be part of the sodium release region. The structures also revealed the pentamerization of KR2 and the binding of sodium ions at the interface [49, 50].

NaR holds significant value for application as a next-generation optogenetics tool with its variants that preferentially pump potassium, another ion that triggers the neuronal responses. Potassium preferential pumps were achieved by manipulating Gly263 or Asn61 residues in the ion-uptake cavity that affect the selectivity of the pump. Furthermore, Kato *et al*. have reported that potassium-pumping KR2 variants presumably manipulate the mammalian neurons and behavioral assays in a nematode [50]. The importance of the three residues of the NDQ motif and their cooperative operation was also demonstrated by studying the molecular properties of wild-type KR2 and its mutants by altering the functional residues. A pivotal role in binding sodium ion of Asn112 was reported by a study, wherein this residue was replaced with 19 different amino acids, and Gln123 contributed to the optimization of the kinetics of sodium-ion uptake and release [51, 52].

Additionally, the mechanism for the rapid photoreaction of KR2 was elucidated via femtosecond time-resolved absorption study or femto- to submillisecond transient stimulated Raman spectroscopy [53, 54]. Moreover, extensive classical and quantum molecular dynamics simulations of transient photocycle states revealed the molecular mechanism for sodium pumping by presenting the electrostatic transitions by internal conformational dynamics and proton transfer reactions [55]. These intensive studies on the molecular mechanism of NaR may provide a basis for the rational design of NaR with optogenetic applications.

## NTQ Motif-Containing Chloride Pump Rhodopsins

Chloride-pumping rhodopsins are found in Archaea and Bacteria living in highly saline habitats, and the presence of these pumps is reported in various marine bacteria inhabiting a broad range of environments [56]. In proton pumps, the proton acceptor and donor amino acids (amino acid residues 85 and 96 in BR) that protonate the retinal Schiff base are conserved as carboxylic amino acids, Asp or Glu [2, 57]. These two residues along with Thr 89 form a highly conserved motif, DTD or DTE, in BR or PR, respectively. HR has the TSA motif at the corresponding position. The single amino acid change in BR (D85T) resulted in the conversion of a proton pump to a chloride pump [58]. The TSD motif was found in cyanobacterial HR, and the single amino acid replacement (T74D) successfully turned the inward chloride pump into an outward proton pump [59].

Yoshizawa *et al*. characterized the first non-archaeal inward chloride pump from the flavobacterium *N. marinus* S1-08 and termed it ‘ClR.’ The function of chloride ion translocation was demonstrated, and the phylogenetic analysis revealed that ClR belongs to a phylogenetic lineage distinct from archaeal HR. In comparison to the TSA motif in HR, ClR has an NTQ motif in its key amino-acid residues that might be involved in chloride ion conductance [16]. The crystal structure of ClR found two chloride-binding sites at the Schiff base and on a cytoplasmic A-B loop, and the chloride-pumping mechanism was revealed along with mutational analyses. Although some features including the binding of a chloride ion at the protonated Schiff base of ClR suggest that ClR might use a chloride transport mechanism similar to that used by HR, the differences represented by key amino-acid residues suggest that these pumps convergently evolved [21]. In ClR, none of the residues involved in chloride ion binding in HR is conserved, and most of the amino-acid residues constituting the internal cavities of ClR differ from those of HR [21]. Spectroscopic studies on chloride pumps also support the differences between ClR and HR. *Fulvimarina* rhodopsin (FR) is another eubacterial ClR with the NTQ motif, which was reported from the alphaproteobacterium *Fulvimarina pelagi*. A chloride ion is accepted by FR on the formation of the O-intermediate, whereas the O-intermediate of HR disappears on acceptance of a chloride ion. This suggests that FR has another chloride ion-binding site near the extracellular side [60]. Furthermore, the spectroscopic study of ClR also revealed that chloride binding affinity is considerably weaker than that of HR [60, 61].

Notably, ClR shares higher sequence identity with NaRs than HRs. Structural studies of ClR indicate that its architecture is similar to that of NaR than that of HR despite the opposite transport directions and charges of the ion molecules transported [21, 62]. The retinal conformation of ClR around the Schiff base is different from that of HR and more similar to those of NaR and XR. The amino-acid residues comprising the internal cavity are highly conserved between ClR and NaR, and they share the 3 omega motif [21].

## Interchangeability of Omega Rhodopsins and Implications in Evolutionary Engineering

Inoue *et al*. attempted functional interconversion between omega rhodopsins to infer evolutionary relatedness among them [63]. As mentioned above, omega rhodopsins possess the conserved functional determinants, that is, DTE, NDQ, and NTQ motifs for H^+^, Na^+^, and Cl^-^, respectively, in helix C. The substitutions in characteristic motifs resulted in positive functional conversions in the case of Na^+^ → Cl^-^ and Cl^-^ → H^+^, whereas the rest of the conversions (H^+^ → Na^+^, H^+^ → Cl^-^, Cl^-^ → Na^+^) remained unsuccessful. Such asymmetric interconversion suggested that the proton pump is the common ancestor of the three rhodopsins, from which the chloride pumps emerged, followed by sodium pumps. The ancestral function was retained even by limited mutagenesis, whereas the gain of a new function cannot be easily reproduced [63]. Furthermore, the substitution of a single amino acid that presumably prevents the carotenoid binding in NaR led to the ability to bind strongly to carotenoid molecules that function as a light-harvesting antenna. This is another example supporting the case that lost functionality in the evolutionary process can be restored by site-directed mutagenesis [64].

Moreover, artificial evolution studies to extend ion or light specificity have been conducted using omega rhodopsins. Successful NaR variants obtained by manipulating residues in the ion-uptake cavity that translocate potassium ions were previously described [49, 50]. Similarly, tuning the same amino acids, Asn61 and Gly263 at the cytoplasmic surface in NaR, turned its variant to permeate Cs^+^ as well as other monovalent cations [65]. Recently, a 40-nm red-shift in the absorption wavelength of NaR was achieved by controlling the polarity of amino-acid residues (P219T/S254A) around the retinal chromophore without impairing its sodium-transport efficiency. Collectively, with the tuning approach, a natural sodium pump with red-shifted absorption, which lacks Pro at the position of P219, was identified from a bacterium living in a solar saltern. Rhodopsins with longer-wavelength light will see expanded use as optogenetics tools due to their low phototoxicity and high tissue penetration [66].

## Evolutionary Relationship Between Omega Rhodopsins and Other Rhodopsins

Although type I and type II rhodopsins share the noticeable structural similarities of possessing the seven-transmembrane α-helical architecture and an internal pocket in which the chromophore retinal is bound, these two types of rhodopsins differ in their sequences and structural details. Moreover, they are also taxonomically distinct and found in evolutionarily distant organisms. Type I rhodopsins that often function as light-driven ion pumps have been found in both prokaryotes and eukaryotes so far, whereas type II rhodopsins are found only in higher animals and classified into the G-protein coupled receptor (GPCR) superfamily since they transduce the light signal to linked G protein [1, 67]. Whether these two protein superfamilies diverge from an ancient common ancestor or converge on the same protein fold and the covalent linkage to the retinal from independent origins remains under debate [67, 68]. Mackin *et al*. suggested that neither the observed rhodopsin fold nor the conserved retinal linkage is necessary for BR photosensitive function based on their empirical test using BR mutants, which implies that type I and II rhodopsins presumably share an ancestor [67]. Furthermore, they proposed that the first type II rhodopsin arose from a descendant of class E GPCRs, cAMP receptors, and thereafter, type I rhodopsins evolved from this type II rhodopsin and underwent relatively rapid sequence changes resulting from loss of G-protein interactions and gain of novel proton-pumping or sensory function [67].

After solving the high-resolution structure of NaR, Shalaeva and colleagues revealed that NaR and sodium-dependent GPCRs share the striking similarity of their sodium-binding sites through structural superposition [68]. Based on the phylogenetic position of NaR between other type I rhodopsins and class A GPCRs, they speculated its proximity to the common ancestor of both superfamilies, which apparently contained a sodium-binding site and presumably was a light-driven sodium export pump. A single aromatic residue (Trp215 in NaR) in the 6th helix turned out to be highly conserved in visual rhodopsins and most type I rhodopsins. It functions in rotation or tilting of the helix, and it might be involved in signal transduction via interacting with ligand in type II rhodopsins and ion translocation by opening a conduit in type I rhodopsins, respectively. Shalaeva *et al*. suggest that the ancestors of GPCRs originated from a sodium-translocating rhodopsin that lost the retinal-binding ability, and re-stabilized by paving the way to the emergence of the sodium-binding site. The reacquisition of retinal by GPCR yielded a visual rhodopsin [68].

The biosynthetic pathway for retinal and carotenoids could be associated with rhodopsin evolution as the key cofactors of rhodopsins. For example, compared to the proteobacterial strains with PR possessing a minimum set of genes for retinal biosynthesis, the flavobacterial strains having NaR or ClR encode an additional *crtZ* gene that converts β-carotene to zeaxanthin [16].

## Ecophysiological Roles of Omega Rhodopsins

Mechanistic evidence on omega rhodopsins has been accumulating rapidly via biochemical studies on photochemical properties and ion specificities and their successful structural analyses, whereas their physiological functions and roles in cellular processes and ecological consequences remain largely unexplored.

It is widely accepted that proton pumps, particularly PR, which is the most abundant phototrophic protein in oceanic surface waters, play a significant role in providing alternative energy generation and metabolic strategies under oligotrophic conditions [69]. The roles of bacterial sodium and chloride pumps in marine microbial ecology are to be determined. Since sodium and chloride are the two most predominant dissolved ions in seawater, their pumps may help in maintaining osmotic balance [69]. They can be more productive if they function in generating energy via a chloride or sodium chemiosmotic potential. Some extremophilic archaea and marine bacteria use the sodium-motive force to drive energy transmission [70]. A respiration-dependent primary sodium pump directly couples Na^+^ translocation to a chemical reaction, or a Na^+^/H^+^ antiporter transforms the H^+^ gradient generated by primary proton pumps into a Na^+^ gradient [71].

In numerous genomes, sodium or chloride pumps coexist with the PR family of proton pumps, and some photo-responsible studies on cell growth or gene expression have been conducted. *Dokdonia* sp. PRO95 having NaR and PR has shown no growth advantage in the light compared to the dark [72]. *N. marinus* S1-08 with three different ion-pumping rhodopsins exhibited light-enhanced growth at low carbon concentration. All three rhodopsins in *N. marinus* S1-08 have very similar absorption spectra, suggesting that they can function simultaneously in the same light field and there could be a functional coupling [16]. In contrast, another Nonlabens strain YIK11, which also contains PR, NaR, and ClR, did not exhibit the light-stimulated growth either in a nutrient-limited or enriched medium, although the gene expression level of the three rhodopsins was higher under illumination [73].

Microbes with omega rhodopsins have been isolated from various environments including freshwater, seawater, halophilic lakes, and salterns. Recruitment of metagenomic reads revealed that the occurrence of NQ rhodopsin-carrying prokaryotes reaches about 17% in a hypersaline microbial mat sample comprising approximately 90 practical salinity units [15]. The metagenomics analysis also verified the existence of XR- and NaR-family omega rhodopsins in Antarctic desert hypolithic communities. It was the first observation of rhodopsins from the terrestrial environment, and they are presumed to exist in ubiquitous species in hypolithons and to adapt well to this environment [74].

The ecological roles of ActR in actinobacteria have been characterized mainly in a ubiquitous freshwater actinobacterial lineage, acI. All retinal biosynthesis pathway genes were transcribed, and ActR was most highly transcribed in a eutrophic lake [40]. A metatranscriptomic study of acI also reported the high expression of ActR and some transport proteins [75]. Notably, the expression level of ActR from the acI lineage in freshwater is not directly light-driven, and it is rather constitutive circadian-minimized at dusk and maximized at dawn [76].

In conclusion, microbial rhodopsins are distributed among microbes belonging to various taxa, spanning a wide range of different characteristics [69]. Among these, we reviewed the studies on rhodopsins with the 3 omega motif in terms of their functional diversity, physiology in the cell, and evolutionary relationship. The reason for the existence of this motif was not clearly elucidated, but we speculate that it may contribute to the structural stability and arrangement of rhodopsins. The presence of this motif may have contributed to the functional diversification of rhodopsins of this class including the ion specificity and the absorption spectrum. Further biochemical research on the 3 omega motif and physiological studies will be required to reveal the importance of omega rhodopsins in evolutionary relationships among rhodopsin (super)families.
